# Systematic profiling of alternative splicing signature reveals prognostic predictor for cervical cancer

**DOI:** 10.1186/s12967-019-02140-x

**Published:** 2019-11-19

**Authors:** Yue-Xin Hu, Ming-Jun Zheng, Wen-Chao Zhang, Xiao Li, Rui Gou, Xin Nie, Qing Liu, Ying-Ying Hao, Juan-Juan Liu, Bei Lin

**Affiliations:** 1 Department of Obstetrics and Gynecology, Shengjing Hospital Affiliated to China Medical University, Shenyang, Liaoning 110004 China; 2 Department of Obstetrics and Gynecology, University Hospital, LMU Munich, Marchioninistr. 15, 81377 Munich, Germany

**Keywords:** Alternative splicing, TCGA, Whole genome level, Cervical cancer, Prognosis

## Abstract

**Aim:**

Cervical cancer is a common malignant carcinoma of the gynecological tract with high morbidity and mortality. Therefore, it is crucial to elucidate the pathogenesis, prevention, diagnosis and prognosis of cervical cancer by searching for the involved key genes.

**Method:**

In this study, the alternative splicing (AS) events of 253 patients with cervical cancer were analyzed, and 41,766 AS events were detected in 9961 genes. Univariate analysis was performed to screen prognostic AS events. Kyoto Encyclopedia of Genes and Genomes (KEGG) enrichment analysis was used to identify the pathways in which these AS events were involved.

**Results:**

We found that exon skip (ES) is the main AS event in patients with cervical cancer. There was pronounced consistency between the genes involved in overall survival and those involved in recurrence. At the same time, we found that a gene may exhibit several different types of AS events, and these different AS events may be related to prognosis. Four characteristic genes, HSPA14, SDHAF2, CAMKK2 and TM9SF1, that can be used as prognostic markers for cervical cancer were selected. Conclusion: The importance of AS events in the development of cervical cancer and prediction of prognosis was revealed by a large amount of data at the whole genome level, which may provide a potential target for cervical cancer treatment. We also provide a new method for exploring the pathogenesis of cervical cancer to determine clinical treatment and prognosis more accurately.

## Background

Cervical cancer is one of the most common malignant gynecological tumors, with an incidence rate second to only breast cancer and the third highest mortality rate following breast and lung cancers [1]. Approximately 470,000 people suffer from this disease worldwide, with 233,000 patients dying from it each year [2]. In developing countries, the incidence and mortality of cervical cancer are on the rise. China has approximately 150,000 new cases of cervical cancer each year, and the incidence tends occur in younger people [1, 2]. Although the relationship between persistent high-risk human papillomavirus (HPV) infection and cervical cancer has been confirmed, only 1% to 3% of the population with HPV infection develop cervical cancer. HPV infection is a necessary factor for the occurrence of cervical cancer and plays an important role in the process of infection and cancer. The three-level prevention strategy for cervical cancer has effectively reduced morbidity and mortality, yet it remains a serious threat to women’s health. Therefore, it is crucial to elucidate the pathogenesis, diagnosis, treatment and prognosis of cervical cancer by searching for the involved key genes and to determine their roles in cervical cancer transformation and progression. At the same time, these genes also provide potential targets for drug action for developing targeted therapeutics.

Protein diversity plays a significant role in the regulation and functional complexity of eukaryotic cellular processes, and alternative splicing (AS) events greatly increase the phenotypes of eukaryotic genes and the diversity of protein function [3]. Selective pre-mRNA splicing is a universal mechanism to produce mRNA isomers using a limited set of genes. Splicing complexes recognize intron–exon junctions by identifying loci with characteristic markers on a mRNA molecule, then perform intron excision and finally connect the adjacent exons. In other words, AS is a posttranscriptional process that can produce different mRNAs from a single gene. Splicing patterns are closely related to the occurrence of human diseases [4, 5]. The influencing factors of AS events include CIS action elements, splice site consistency, conservative sequences and trans-action factors, any change in which can lead to splicing abnormalities.

The Cancer Genome Atlas (TCGA) project, a public database that was completed by the National Cancer Institute (NCI) and the National Human Genome Research Institute (NHGRI) [6], aims to map the genome variants of all human cancers by using large-scale genome sequencing techniques. With the advantage of high-throughput RNA sequencing, TCGA has been a great source for the study of AS events [7]. SpliceSeq is a Java program that can provide information on each exon and splice site. Ryan et al. expanded the calculation of SpliceSeq and obtained potential events for 33 types of cancer-specific AS events [8]. Our study systematically analyses all AS events in cervical cancer for the first time using the TCGA database and describes several newly discovered selective splicing events related to survival. It also explores the genes associated with cervical cancer and provides a new way to explore the pathogenesis of cervical cancer, which will guide clinical treatment more accurately and help determine patient prognoses.

This study included 253 cervical cancer cases that were shared in the TCGASpliceSeq database and RNA-seq expression spectra, from which 19,754 genes with expression values were obtained. There are seven types of AS events for cervical cancer, including exon skip (ES), mutually exclusive exons (ME), retained intron (RI), alternate promoter (AP), alternate terminator (AT), alternate donor site (AD), and alternate acceptor site (AA). The data distributions of all encoded genes from the samples regarding the seven different types of AS events were analyzed, and ES events were found to be the main type of AS event in cervical cancer. We then used an R package [9] for univariate analysis, and the results showed that most ES events were not associated with prognosis, but approximately 10% of AT events were significantly correlated with recurrence in cervical cancer. By constructing a network of genes that have significant roles in AS associated with prognosis, we found that most genes have protein interactions. KEGG enrichment analysis revealed the selective splicing pathways associated with prognosis. To observe whether the relationship between gene expression and prognosis in AS events significantly correlated with prognosis, univariate analysis of each gene was performed using TCGA RNA-seq expression profile data. Using Pearson correlation analysis, four recurrence-related genes were screened from 46 genes associated with overall survival: HSPA14, SDHAF2, CAMKK2 and TM9SF1. These four characteristic genes were used to construct a multivariate survival model, and the results showed that the four genes may be used as prognostic markers of cervical cancer. This discovery may provide a new idea for the early diagnosis and treatment of cervical cancer.

## Methods

### Data download and preprocessing

RNA-seq variable splicing event data for cervical cancer were obtained from the TCGASpliceSeq database and included 256 samples, three of which were normal samples. The RNA-seq expression profiles of cervical cancer were obtained from the TCGA database and included 309 samples, three of which were normal samples. We downloaded all clinical follow-up information provided in TCGA for a total of 307 samples with clinical follow-up information. The RNA-seq expression profile data set in fragments per kilobase per million fragments mapped (FPKM) was downloaded and converted into trusted platform module (TPM) data. At the same time, the IDs were converted using the GRCh38.p2 version of GENCODE’s genome file to obtain each encoded protein’s gene, and 253 samples of TCGASpliceSeq and RNA-seq were selected for further study.

### Screening for prognostic AS events in cervical cancer

TCGASpliceSeq is a database based on TCGA RNA-seq data that uses SpliceSeq tools to analyze mRNA splicing patterns with seven types of selective splicing events: ES, ME, RI, AP, AT, AD and AA. We analyzed the distributions of all encoded genes in each of the seven different types of AS events in cervical cancer. Different AS events in genes led to diversity in outcomes, and changes in gene expression affected survival time. The ***Survival.R*** package was used for the univariate analysis of different AS events. A gene with significant p < 0.05 was selected as an AS event with different prognoses.

### Prognostic selective splice event gene type analysis

AS events affect gene translation and protein diversity, so we selected AS events related to prognosis and analyzed the distributions of genes for different types of AS events related to prognosis. To determine whether AS events can be used as prognostic factors, we selected the most significant genes using multivariate regression model analysis and observed their effects on prognosis.

### Gene interactions and functional analysis in different types of prognostic AS events

To observe the association between significant genes in different types of AS events and prognosis, we mapped the genes to the Search Tool for the Retrieval of Interacting Genes/Proteins (STRING) database using a score greater than 0.4 to obtain the interaction relationship of these genes and used Cytoscape [10] for visualization. Then, we used the ***clusterprofiler.R*** package to conduct KEGG enrichment analysis on the AS genes significantly correlated with prognosis in each type of AS event and observed the pathways enriched by these genes.

### Relationship between gene expression and prognosis in AS events

To determine the relationship between gene expression and prognosis in AS events that significantly correlated with prognosis, we used TCGA RNA-seq expression profile data to perform univariate analysis survival of each gene separately.

### Characteristic gene selection and construction of a prognostic model

Genes with Pearson correlation coefficients greater than 0.2 or less than − 0.2 were selected as prognostic feature genes to construct prognostic models suitable for cervical cancer. The classification of prognosis by these prognostic characteristic genes were observed at the level of AS events and expression profiles.

## Results

### ES is the main selective splicing event in patients with cervical cancer

**Fig. 1:**
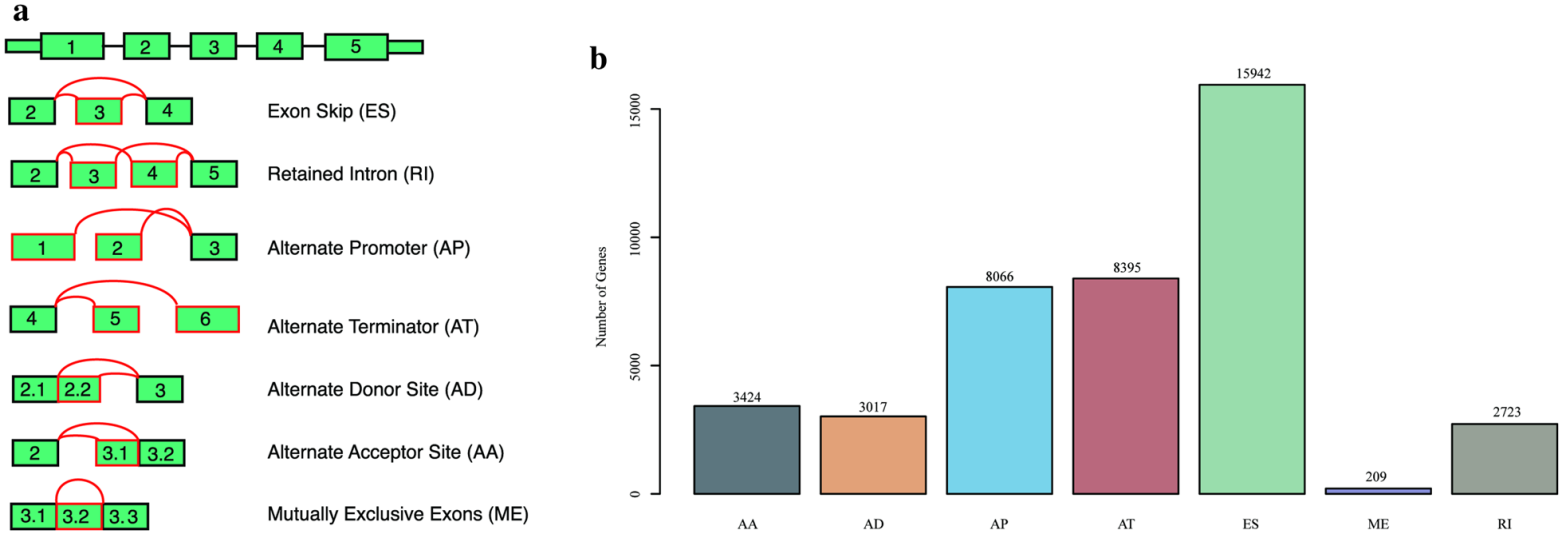
The distribution of seven AS events in cervical cancer. **a** Seven different AS event types. **b** Number of AS events of each of the seven types

We counted all AS events in 253 cancer samples, as shown in Additional file 1: Table S1; the seven AS events are shown in Fig. 1a. We detected 41,766 AS events in 9961 genes, of which the seven types of AS events were distributed as shown in Fig. 1b. A single gene might display several types of mRNA AS events, and we found that nearly one-third of all AS events were ES events.

### Pronounced consistency between the genes involved in overall survival and the genes involved in recurrence

**Fig. 2:**
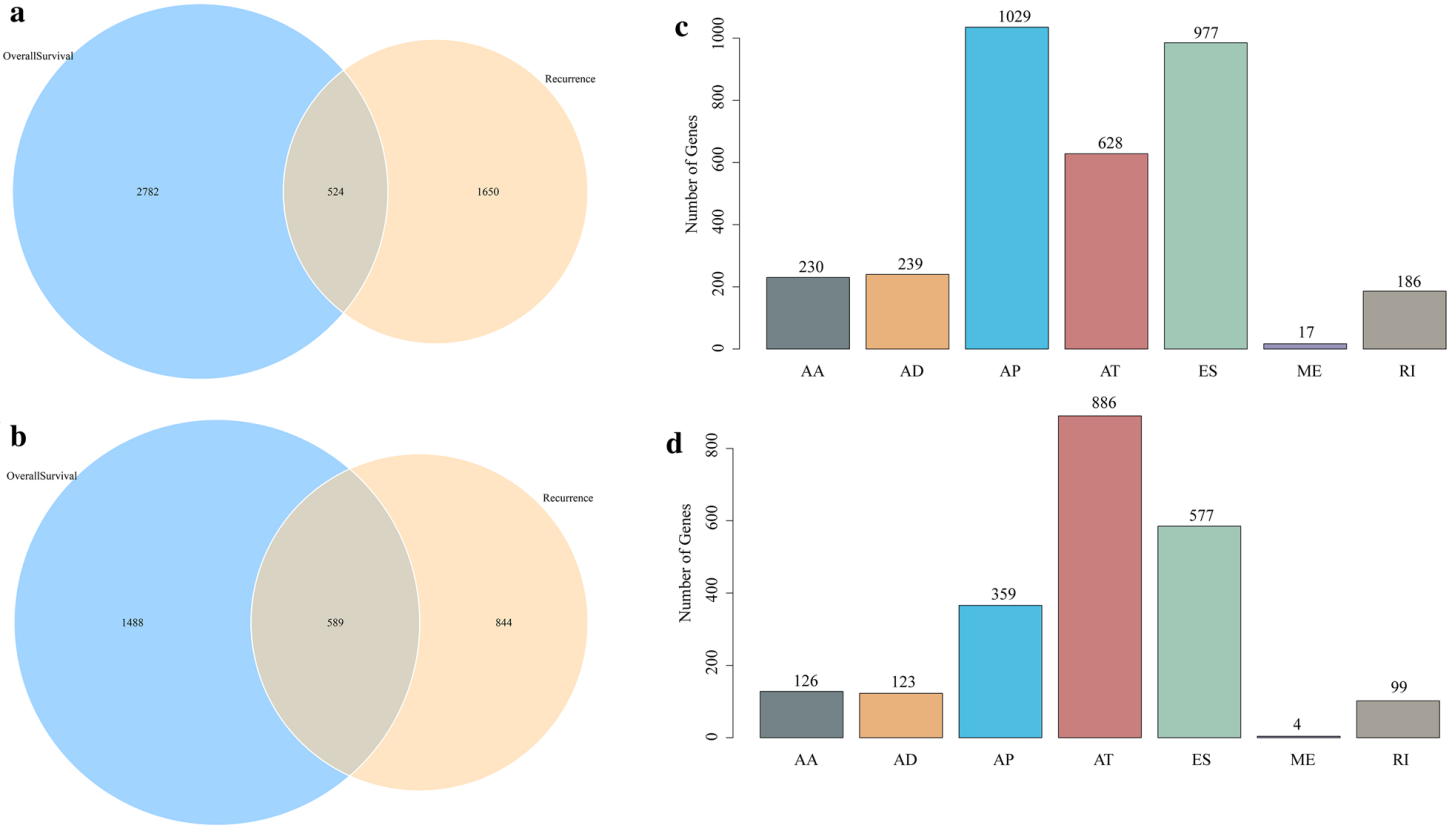
Analysis of relationship between AS events and prognosis. **a** Venn diagram of AS events associated with overall survival and recurrence. **b** Venn diagram of the gene intersection of AS events significantly related to overall survival and recurrence. **c** Statistical histogram of the seven types of AS events significantly related to overall survival. **d** Statistical histogram of the seven types of AS events significantly related to recurrence

To observe the relationship between AS events and prognosis, we integrated clinical follow-up data as shown in Additional file 2: Table S2. A total of 41,766 AS events were analyzed by univariate survival analysis to observe the relationship between these AS events and the prognosis of patients with cervical cancer. By selecting p < 0.05, we obtained a total of 3306 AS events that were significantly related to survival and 2077 genes. There were 2174 AS events significantly associated with the recurrence of cervical cancer, including 1443 genes (Additional file 3: Table S3). There were 524 intersections of AS events that were significantly related to overall survival and recurrence (Fig. 2a) that contain a total of 589 overlapping genes (Fig. 2b). This shows that there is a pronounced consistency between the genes involved in overall survival and the genes associated with recurrence. The statistics of the AS events significantly related to overall survival are shown in Fig. 2, which shows that the largest proportion of ES events are reduced in relation to recurrence. In contrast, AP and AT events increased, and among the AS events associated with recurrence, AT events occurred the most (Fig. 2d), indicating that most ES events were not associated with prognosis, while approximately 10% of AT events were significantly associated with recurrence of cervical cancer.

### A gene may have multiple AS events of different types

**Fig. 3:**
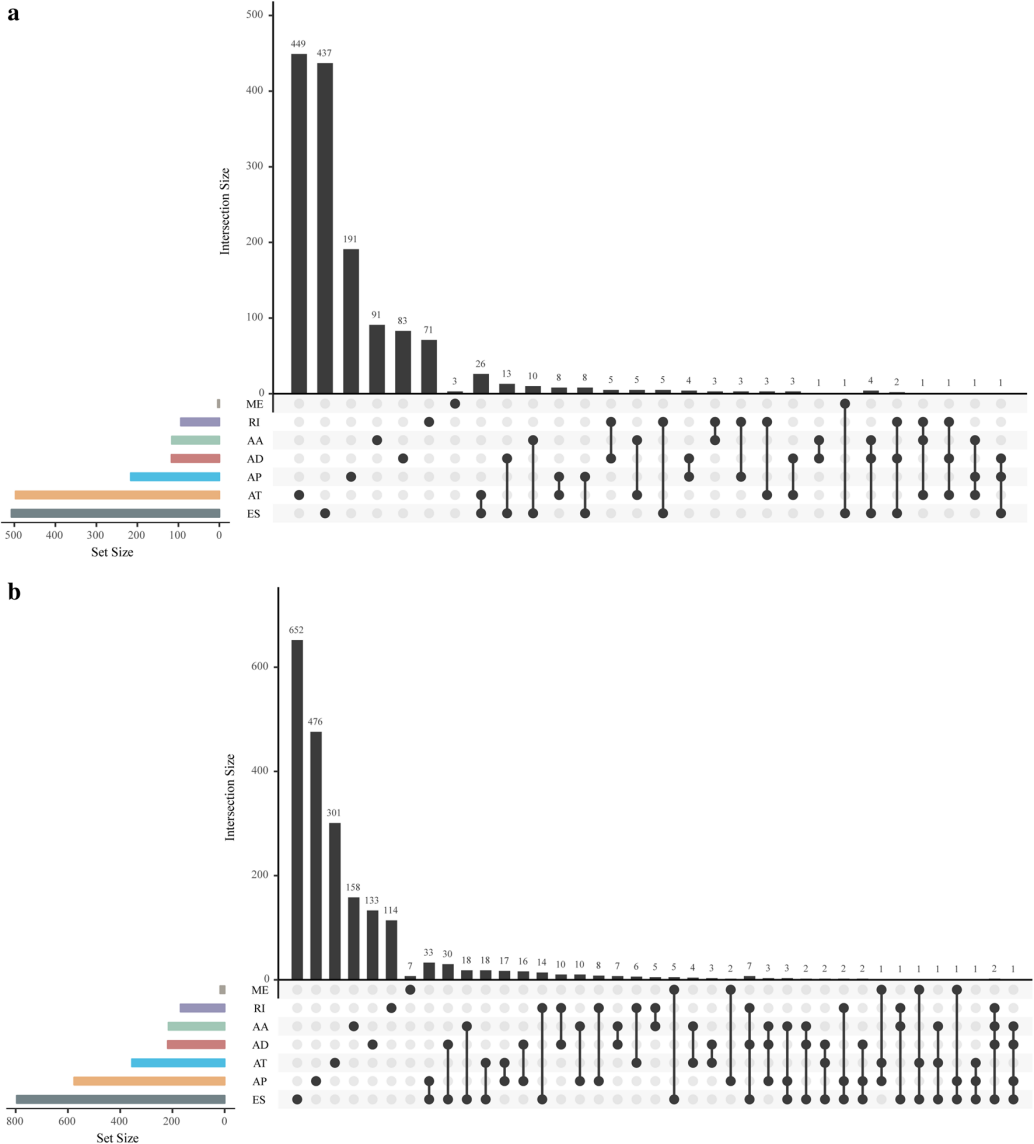
Prognosis-related AS event gene type analysis. **a** Distributions of the seven different AS events that are significantly associated with recurrence. **b** Distributions of the seven different AS events that are significantly associated with overall survival

Because AS events affect gene translation and subsequent protein diversity, we selected AS events related to prognosis to analyze the distributions of the genes involved in these events. As shown in Fig. 3a, b, a gene can be seen in several different types of AS events, and each of these AS events may be related to prognosis.

### AS events can be used as a new prognostic classification for cervical cancer

To observe whether an AS event can be used as an independent prognostic factor for overall survival, the multivariate Cox regression model was applied to the seven types of survival-related AS events. According to the results of the univariate Cox regression model, the first 15 genes with the most significant AS event p value per type were selected. Multivariate Cox analysis models were carried out according to the percent spliced in (PSI) value of the AS events of these 15 genes.

**Fig. 4:**
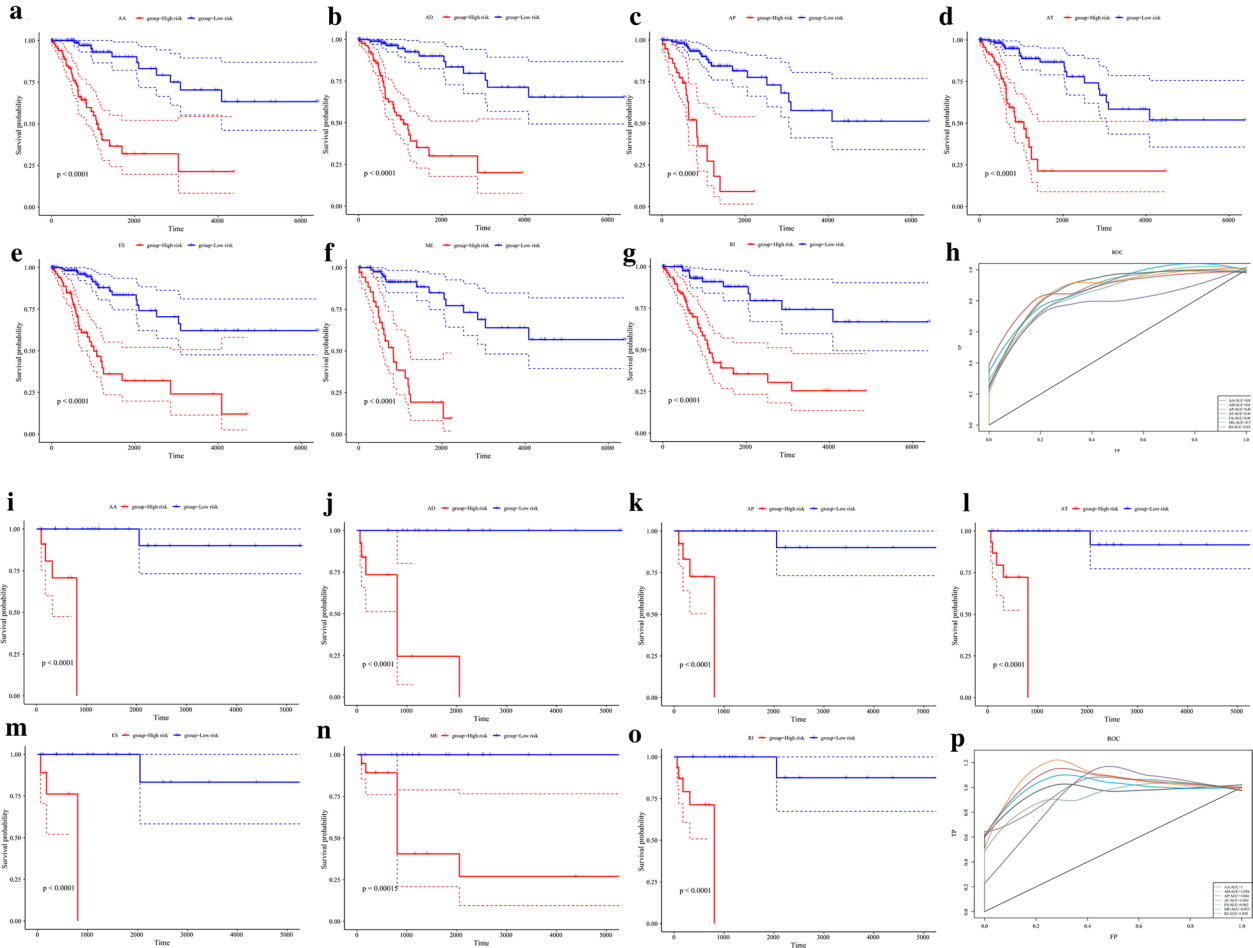
Cervical cancer AS event prognostic factor analysis. **a**–**g** Kaplan–Meier curves of prognostic predictors associated with overall survival constructed with each type of AS event for cervical cancer patients. The red line indicates a high-risk group, while the blue line indicates a low-risk group. **h** ROC curves with AUCs for overall survival constructed with each type of AS event in cervical cancer. **i**–**o** Kaplan–Meier curves of prognostic predictors associated with recurrence constructed with each type of AS event for cervical cancer patients. The red line indicates a high-risk group, while the blue line indicates a low-risk group. **p** ROC curves with AUCs for recurrence constructed with each type of AS event in cervical cancer

The results are presented in Fig. 4a–g, which shows that the seven predictors built with different types of AS events have considerable power in distinguishing favorable or poor outcomes for cervical cancer patients. As shown in Fig. 4h, the type of AS event can classify patient overall survival; the performance of the AP and AT models are optimal for overall survival (area under the curve (AUC) = 0.89), followed by the ES model (AUC = 0.86).

Then, we used multivariate Cox regression models to determine whether the seven types of AS events could be used as independent prognostic factors for predicting recurrence in patients with cervical cancer. As presented in Fig. 4i–o, the seven predictors built with different types of AS events showed considerable power in distinguishing favorable or poor outcomes for cervical cancer patients.

Regarding AS events associated with recurrence (Fig. 4p), the AT, AA, AD, AP and RI models performed optimally, with AUCs close to 1, suggesting that AS events could be used as a new method of prognostic classification.

### Forest map analysis

**Fig. 5:**
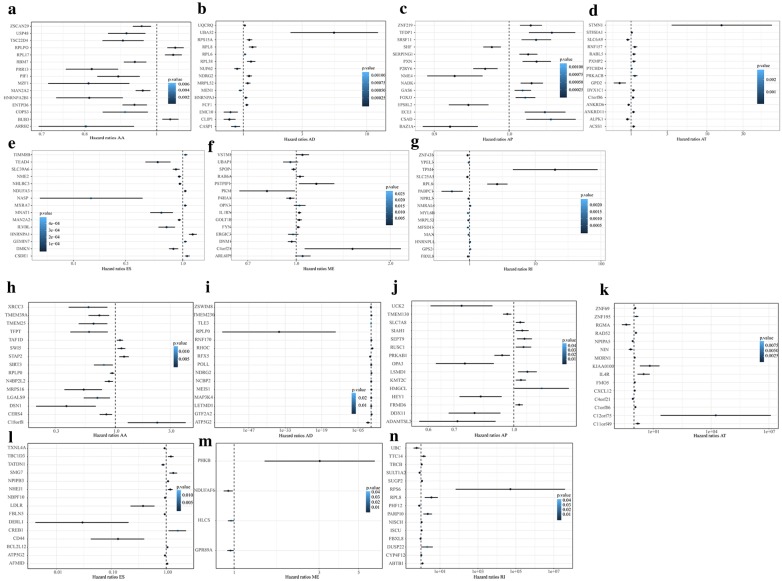
Forest plots of survival-associated AS events in cervical cancer. **a**–**g** Hazard ratios of the top 15 overall survival-associated AA, AD, AP, AT, ES, ME and RI events, respectively. P values are indicated by the color scale on the right side. **h**–**i** Hazard ratios of the top 15 recurrence-related AA, AD, AP, AT, ES, ME and RI events, respectively

The most significant top 15 genes in each type of AS event were selected for forest map analysis (Fig. 5). Most of the survival-associated AA events were favorable for prognosis (HR < 1) (Fig. 5a, h), which is consistent with recurrence risk, and the AP events were almost identical in recurrence and overall survival.

### Gene interaction networks and functional analysis of the different types of AS events

**Fig. 6:**
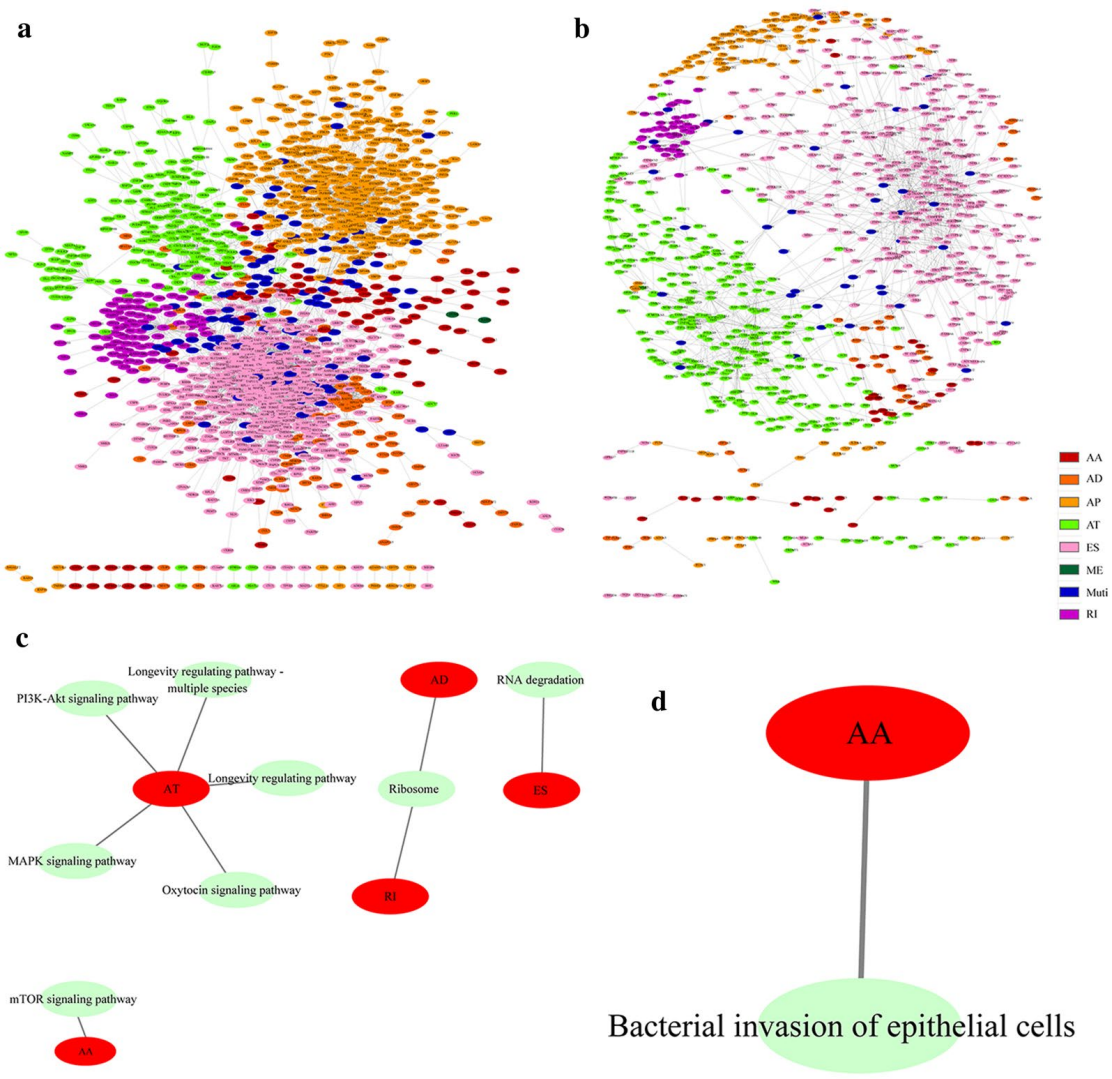
Gene interaction networks of different types of AS events significantly related to prognosis. **a** Seven different gene interaction networks in AS events that are significantly associated with overall survival, and seven different gene interaction networks in AS events that are significantly associated with recurrence. **b** A network of the seven different variable splicing events that are significantly associated with relapse. **c** KEGG enrichment analysis of seven different AS genes significantly related to overall survival. **d** KEGG enrichment analysis of seven different AS genes significantly associated with recurrence

As mentioned previously, one gene might have more than one AS event. To observe the relationship between different types of genes in AS events that are significantly related to prognosis, we mapped the genes to the STRING database to obtain the interaction of these genes using a score greater than 0.4. Cytoscape was used to visualize the results shown in Fig. 6a, b. AD and RI show most of the interactions, and most of the genes in AS events related to prognosis have protein interactions. To observe the function of genes in different types of AS events that were significantly associated with prognosis, KEGG enrichment analysis was performed for each type of gene significantly related to prognosis. The results are shown in Fig. 6c, d. We found that the enrichment of these genes is related to multiple disease pathways, suggesting that the genes are involved in many biological functions.

### Relationship between gene expression profile and prognosis in AS events

To observe the relationship between gene expression and prognosis in AS events, we used TCGA RNA-seq expression profile data to analyze the survival rate related to each gene and found that 241 AS genes were associated with overall survival. The expression of 159 AS genes was significantly correlated with recurrence. Furthermore, we analyzed the correlation between the 241 genes associated with overall survival and the occurrence of AS events and found that 115 genes were significantly correlated with AS (Pearson p < 0.05). Similarly, 71 of the 159 genes associated with recurrence were significantly associated with AS events, indicating that the AS events of most genes were significantly associated with their expression.

### Characteristic gene selection and construction of a prognostic model

**Fig. 7:**
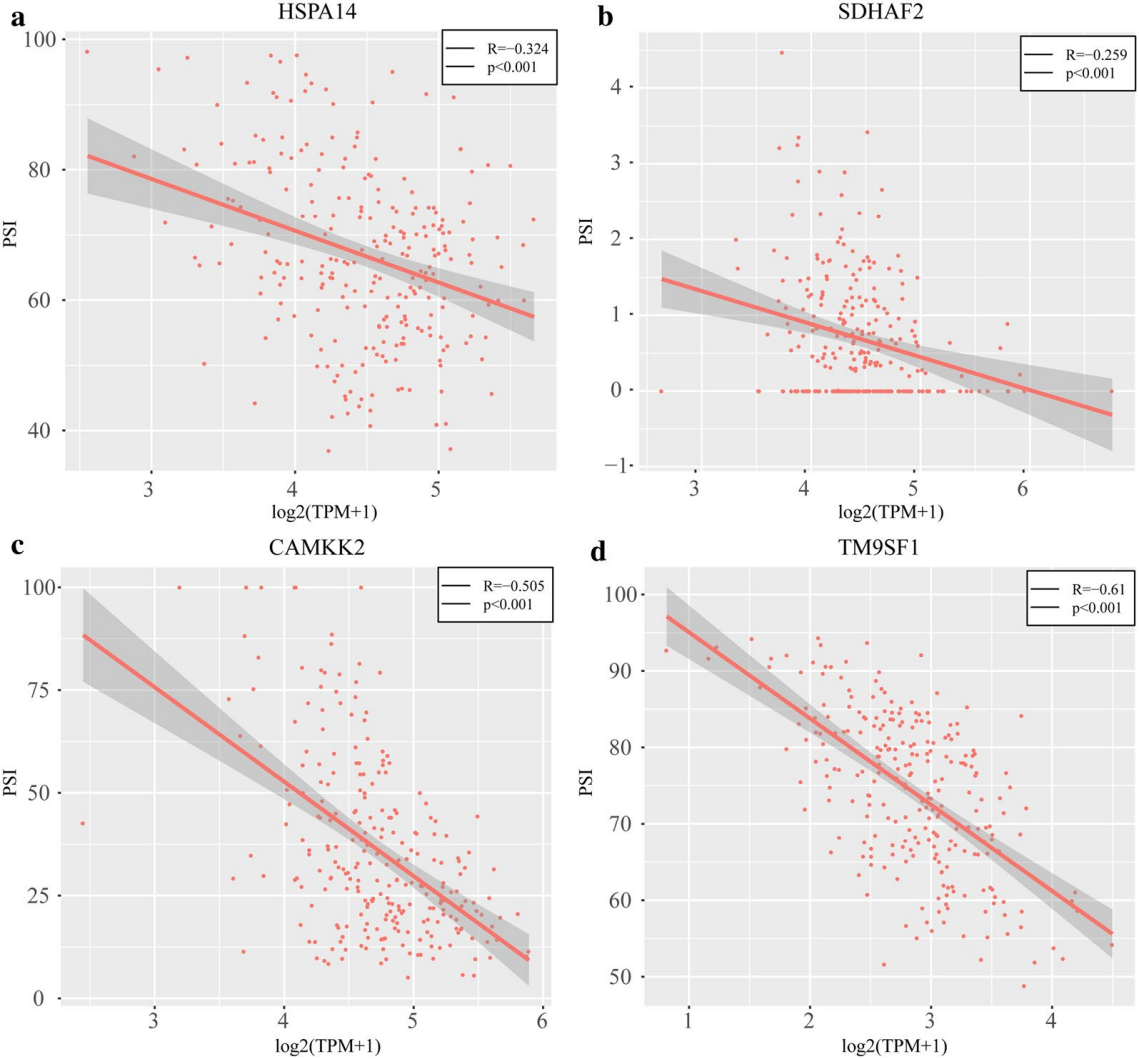
Correlation between AS and transcription levels of the four characteristic genes. **a** Correlation between AS and transcription levels for HSPA14. **b** Correlation between AS and transcription levels for SDHAF2. **c** Correlation between AS and transcription levels for CAMKK2. **d** Correlation between AS and transcription levels for TM9SF1

We chose to study those genes with Pearson correlation coefficients greater than 0.2 or less than − 0.2 for gene expression and AS events. There were 46 genes related to the overall survival of patients with cervical cancer and 82 related to recurrence. Four genes were associated with both overall survival and recurrence: HSPA14, SDHAF2, CAMKK2 and TM9SF1 (correlations with transcriptome levels are shown in Fig. 7). The graph shows that all of these genes have negative correlations between AS and transcription levels.

**Fig. 8:**
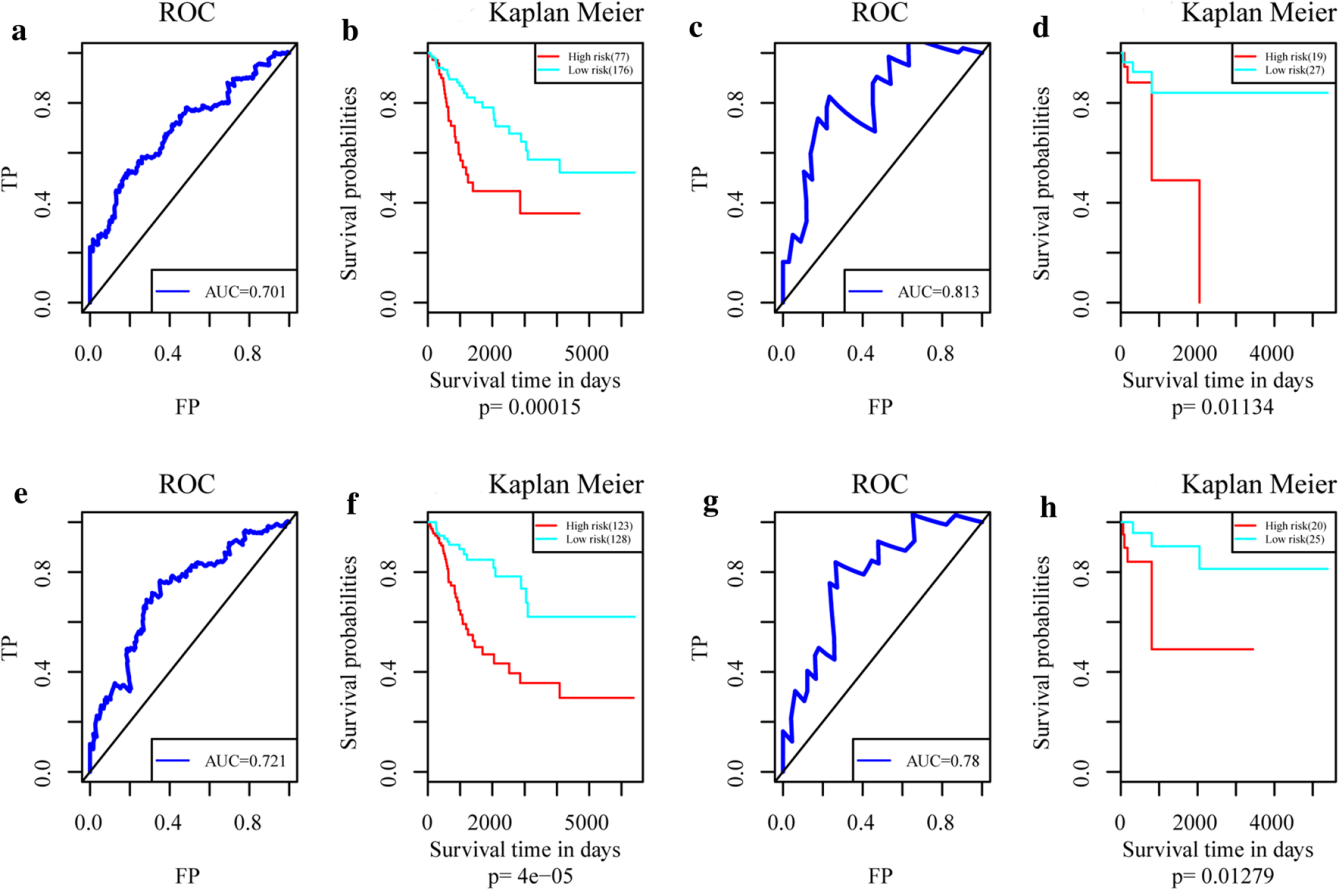
Results of the multivariate survival analysis of the four characteristic genes. **a** AUC curve of the multivariate prognostic model for the overall survival time of AS events. **b** K–M curve of the multivariate prognostic model for the overall survival time of AS events. **c** AUC curve of the multivariate prognostic model for the recurrence of AS events. **d** K–M curve of the multivariate prognostic model for the recurrence of AS events. **e** AUC curve of the multivariate prognostic model for overall survival time at the transcriptional group level. **f** K–M curve of the multivariate prognostic model for overall survival time at the transcriptional group level. **g** The AUC curve of the multivariate prognostic model for recurrence at the transcriptional group level. **h** The K–M curve of the multivariate prognostic model for overall survival time at the transcriptional group level

**Fig. 9:**
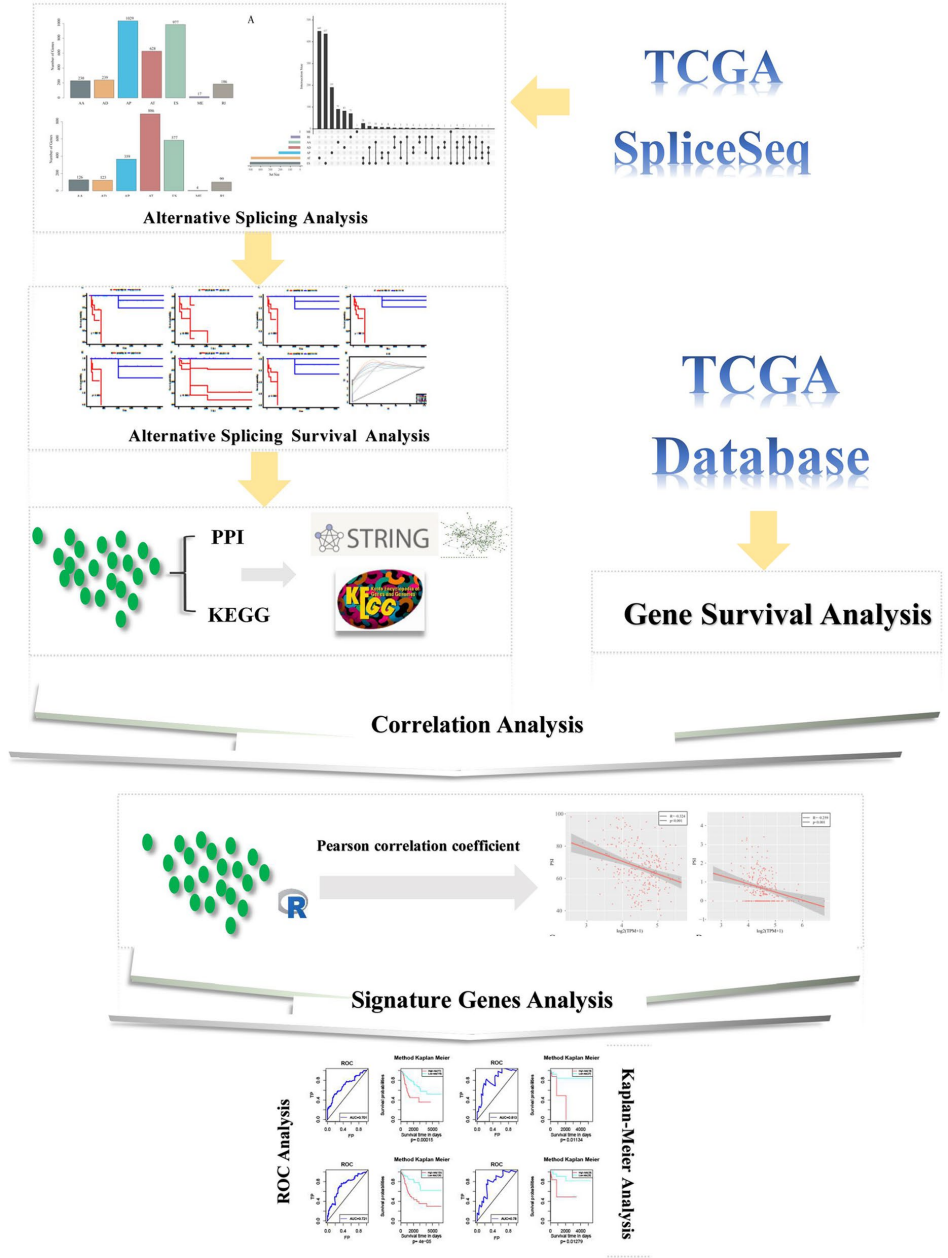
Flowchart of the bioinformatics approach

The four characteristic genes were selected for the construction of a multivariate survival model to classify prognosis regarding AS events and gene expression profiles. The map shows that these four genes have a high prognostic classification effect in both datasets (Fig. 8b, d, f, h), and the AUC value for 5 years is greater than 0.7, with high accuracy (Fig. 8a, c, e, g). These results suggest that these four genes can be used as prognostic markers of cervical cancer. The flow chart of the whole data analysis is shown in Fig. 9.

## Discussion

Variable mRNA splicing is prevalent in eukaryotic cells, and Modrek et al. found that 35–59% of genes in the human genome display AS events [10]. Because AS requires the participation of many factors, an abnormality of any one factor will change the original splicing mode, thus precipitating abnormal or even opposing protein functions, leading to disease occurrence. There is a direct relationship between the occurrence of some hereditary diseases [11] and AS events.

Current studies have confirmed that the abnormal selective splicing of pre-mRNAs is involved in the occurrence and development of cancer. The splicing products of some genes are specifically overexpressed in some tumors, and the isomer RON corresponding to the deletion of exon 11 of the RON gene has strong activity. The abnormal high expression of RON in breast and colon cancers can enhance the ability of cell migration and then affect embryogenesis, histogenesis and tumor metastasis [12]. The splicing product of the CD44 gene CD44v8-10 was significantly upregulated in gastric cancer. Its overexpression directly leads to the enhancement of the metastasis potential of tumor cells [13, 14]. KAI1 is a tumor suppressor gene that inhibits tumor metastasis. The isomer KAI1-SP, which lacks exon 7, is highly expressed in ovarian metastases. Further studies have found that this isomer has a carcinogenic effect. It can also improve the invasive ability of tumor cells [15]. These specifically expressed AS products may become a new target for the treatment of tumors in the future. Although many cancer-specific mRNA isomers have been identified, the understanding of AS events and their functional pathways is still far from being elucidated. The rapid development of high-throughput sequencing and bioinformatics makes it possible to comprehensively explore the AS events of cervical cancer.

In this study, we screened prognosis-related AS events and discussed the relationship between AS events and the prognosis of cervical cancer for the first time using the TCGA database, which provided a new idea for exploring the pathogenesis of cervical cancer to guide clinical treatment and judge prognosis more accurately. In this study, the characteristics of AS events in 253 patients with cervical cancer were analyzed. A total of 41,766 AS events were detected in 9961 genes, of which ES events were the main type, accounting for 1/3 of the total AS events. AT events were the second most frequently occurring, followed by AP events. Subsequently, we performed a univariate survival analysis of 41,766 selective splicing events and found that most of the ES events were not associated with prognosis, but approximately 10% of AT events were significantly associated with recurrence of cervical cancer. At the same time, we found that multiple types of AS events may occur in a gene, and these different AS events may be related to prognosis. The seven types of AS events had high AUC values. For overall survival, the AP model performed the best, followed by the ES model. Among the AS events significantly related to recurrence, the AT and AP models performed best, suggesting that AS events can be used as a new prognostic classification method. By constructing the interaction network of genes in AS events, we found that most of the genes in AS events related to prognosis had protein interactions. To explore the functions of the genes involved in AS events, a KEGG enrichment analysis of the genes was carried out. The results show that AT events are involved in a variety of signaling pathways, including the PI3 K-Akt signaling pathway, MAPK signaling pathway, and oxytocin signaling pathway; AD and RI events are related to ribosomal pathways; and ES events are related to RNA degradation. To observe the relationship between gene expression and prognosis in AS events, univariate survival analysis and Pearson correlation analysis were conducted, and the results showed that the AS events of most genes were significantly correlated with their expression. Subsequently, we selected genes whose Pearson correlation coefficient between gene expression and AS events was greater than 0.2 or less than − 0.2 that were related to both overall survival and recurrence. There were 4 genes in total: HSPA14, SDHAF2, CAMKK2 and TM9SF1. These four characteristic genes were used to construct a multivariate survival model, which had a significant prognostic classification effect on AS events and gene expression profiles; the AUC value for 5 years was more than 0.7, which indicated high accuracy. These results suggest that the four genes can be used as prognostic markers of cervical cancer.

HSPA14 gene translation can produce a heat shock protein, which is expressed in the lens of zebrafish, forms a mammalian ribosomal complex with MPP11, and acts as a Th1 adjuvant to activate dendritic cells [16–19]. The siRNA-mediated inhibition of HSPA4 or HSPA14 could reduce the migration, invasion and transformation activity of NBS1 overexpression cells in vitro. The expression of NBS1 is closely related to the prognosis of patients with advanced head and neck squamous cell carcinoma (HNSCC) [20]. SDHAF2 gene translation can produce a protein that interacts with succinate dehydrogenase (SDH). It has been confirmed that the mutation of the SDHAF2 gene can lead to hereditary paraganglioma-pheochromocytoma type 2. The product of the CaMKK2 gene belongs to the serine/threonine protein kinase family and the Ca2+/calmodulin-dependent protein kinase subfamily. Its main function is to regulate its downstream kinases CaMK1 and CaMK4 to further activate or inactivate related proteins through phosphorylation. In addition, it can also phosphorylate AMPK and participate in the occurrence and development of a variety of diseases [21]. CaMKK2 was significantly upregulated in hepatocellular carcinoma (HCC) and negatively correlated with the survival rate of HCC patients. TM9SF1 is an autophagy-induced gene that has been shown to be associated with Fuchs’ endothelial dystrophy and corneal disease. In summary, most of the above four characteristic genes play an important role in the process of cancer. Cervical cancer is a genetic disease with the participation of a variety of oncogenes and anticancer genes and the synergistic effect of multiple stages and pathways. The results showed that the proto-oncogene Bmi-1 was associated with clinical stage, tumor grade and lymphatic metastasis, and the Hk6 gene was overexpressed in cervical cancer [22, 23]. EGFR, NOTCHI, RHOA and other genes are associated with the lymph node metastasis of cervical cancer [24]. These studies are of great significance in revealing the role of RNA splicing in the occurrence of cervical cancer. An in-depth analysis of the RNA splicing model may indeed reveal new cancer drivers and provide insights into the mechanisms of these pathways. There are still several limitations in our research. First, the number of patients included in TCGA is limited. Second, the mechanisms of the four characteristic genes in cervical cancer are unclear. The abnormal proteins produced by the translation of the characteristic genes of AS events of cervical cancer are very important. In the follow-up study, we will further verify the expression of these characteristic genes and the molecular mechanisms involved in the occurrence and development of cervical cancer.

## Conclusions

In conclusion, these results highlight the value of AS events in the prognosis of cervical cancer and explore the potential regulatory mechanisms of cervical cancer. A large amount of data at the whole-genome level revealed the significance of AS events in the occurrence and development of cervical cancer, especially in the prediction of prognosis, which may provide a potential target for the treatment of cervical cancer.

## Supplementary information

**Additional file 1: Table S1.** Alternative splicing events in 253 cancer samples.

**Additional file 2: Table S2.** Integrated clinical follow-up data of cervical cancer patients.

**Additional file 3: Table S3.** Prognosis-related alternative splicing events of cervical cancer.

## Data Availability

All data in the current study are based on public data available in The Cancer Genome Atlas (TCGA) datasets.

## Abbreviations

KEGG: Kyoto Encyclopedia of Genes and Genomes
HPV: human papillomavirus
ES: exon skip
ME: mutually exclusive exons
RI: retained intron
AP: alternate promoter
AT: alternate terminator
AD: alternate donor site
AA: alternate acceptor site
HSPA14: Heat Shock Protein Family A (Hsp70) Member 14
SDHAF2: Succinate Dehydrogenase Complex Assembly Factor 2
CAMKK2: Calcium/Calmodulin-Dependent Protein Kinase Kinase 2
TM9SF1: Transmembrane 9 Superfamily Member 1
